# Rheumatoid Arthritis in Sickle-Cell Population: Pathophysiologic Insights, Clinical Evaluation and Management

**DOI:** 10.4172/2161-1149.1000225

**Published:** 2017-09-12

**Authors:** Isabel M McFarlane, David J Ozeri, Yair Saperstein, Milena Rodriguez Alvarez, Su Zhaz Leon, Kristaq Koci, Sophia Francis, Soberjot Singh, Moro Salifu

**Affiliations:** 1 Department of Medicine, Divisions of Rheumatology and Nephrology, State University of New York, USA; 2 Department of Medicine, Division of Rheumatology, New York Presbyterian Methodist Hospital, USA

**Keywords:** Sickle cell disease, Rheumatoid arthritis, Vaso-occlusive crises, Pathogenesis, Treatment, Epidemiology

## Abstract

The advent of hydroxyurea and advanced medical care, including immunizations has led to improved survival among patients with Sickle Cell Disease (SCD). This prolonged survival however, introduces a chronic inflammatory disorder, Rheumatoid Arthritis (RA), which presents at a relatively older age and is rarely reported among SCD patients. In this review, we highlight the epidemiological association of SCD-RA and discuss the underlying common pathogenetic mechanisms, such as endothelial dysfunction, the role of inflammatory cytokines and oxidative stress. We also point to the difficulties in ascertaining the clinical diagnosis of RA in SCD patients. Finally, we provide rationale for therapeutic options available for RA and the challenges in the management of these patients with agents that are known to increase the risk of infection and immunosuppression such as steroids, disease modifying anti-rheumatic drugs and biologics.

## Introduction

Professor James Herrick was the first to describe abnormal Red Blood Cells (RBC) in a dental student with severe anemia [1]. However, the recognition that SCD was caused by a molecular defect in hemoglobin came in 1949 through the work of Pauling [2]. Inherited as autosomal recessive, SCD are a group of genetic disorders caused by mutations in the ß-globin gene. The most common form is Hemoglobin S (HbS) in which a single amino acid substitution (from glutamic acid to valine) allows hemoglobin polymerization in conditions of low oxygen. This leads the RBC to dehydrate due to loss of cations and water. The RBCs are unable to maintain their flexibility and shape due to the increased viscosity of the cytosol, and therefore acquire the typical sickle shape [3]. We have learned that the RBC membrane in SCD undergoes iron mediated changes due to precipitation and oxidation of globin polymers leading to a tendency for the sickled RBC to adhere to the endothelium [4,5]. Vaso-Occlusive Crises (VOC) are postulated to occur due to blockage of the blood flow by the sickled red cells which lead to acute chest syndrome, ischemia, stroke, infarcts, pain, and the development of bone marrow degeneration and bony infarcts. The exact prevalence of SCD in Americans is unknown. Hassel in 2010 estimated to be 72,000 to 98,000 cases [6]. The Centers for Disease Control and Prevention estimate that 100,000 Americans are affected by SCD. Survival rates estimates of 43 years for females and 41 years for males, originate from the Registry and Surveillance System project funded by the NIH, a population-based study on SCD that was conducted in the states of California and Georgia from 2004–2008 [7,8].

RA, with a prevalence of 1% in the adult population, is a chronic systemic inflammatory disease characterized by inflammation and proliferating synovitis (pannus). It leads to damage of cartilage and to juxtarticular bone destruction, resulting in joint deformities and functional disability [9]. The EPPPRA project, a prospective epidemiological survey to describe prevalence and clinical aspects of RA in the French West Indies, found a prevalence of RA among Afro-Caribbean to be 0.10% [10].

The exact etiology of RA is not known, although environmental factors (smoking and infection), as well as genetic predisposition, are known to contribute. The genetic basis for RA occurring among African-Americans (AA) is largely understudied. From the studies performed, mostly including Caucasian patients, we learned that Human Leukocyte Antigen-DRB1 (HLA-DRB1), alleles containing the shared epitope, are markers of disease risk and severity. However, HLA-DRB1 has been found in only one third of AA RA patients [11]. Moreover, single nucleotide polymorphisms genome studies revealed differences in the allele frequencies in the Tumor Necrosis Factor receptor (TNFR) genes (TNFRSF1A and TNFRSF1B) between AA (healthy and with RA) and Caucasians, suggesting that these differences could imply higher likelihood to develop RA, influence disease severity and response to biologics [12].

The Consortium for the Longitudinal Evaluation of African Americans with Early Rheumatoid Arthritis (CLEAR) Investigators encountered a higher expression of Interferon ɣ receptor 1 and 2 genes (IFNGR1 and IFNGR2) in peripheral blood cells among AA with RA, higher expression of IFNGR2 was associated with increased radiographic severity; the authors suggested not only a potential pathogenic role of IFN-ɣ in terms of susceptibility and aggressiveness of disease but also its utility as a biomarker for severe radiographic progression [13]. We have also learned that AA with RA sustain early damage in the course of the disease and undergo a radiographic progression similar to that seen in other ethnic groups [14]; this finding is contrary to the previously held notion that RA affected black patients in a less aggressive manner.

## Methods

MEDLINE was searched via PubMed for English-language articles published until May 2017. The search terms were ‘sickle cell disease and rheumatoid arthritis’, ‘sickle cell disease and synovitis’, ‘sickle cell disease and connective tissue disease’, ‘sickle cell disease and inflammatory arthritis’, ‘hemoglobinopathy and rheumatoid arthritis’, ‘hemoglobinopathy and synovitis’, ‘hemoglobinopathy and connective tissue disease’, and ‘hemoglobinopathy and inflammatory arthritis’. Relevant references from selected publications were identified and summarized in this review.

### Sickle Cell Disease and Rheumatoid Arthritis

Rheumatoid arthritis, although prevalent across western countries, has been seldom reported co-existing with SCD. Chronic inflammatory arthritis mimicking rheumatoid arthritis was first reported by Coodley et al. prior to the use of RA serologies. They reported a case of a man with SCD who developed chronic erosive arthritis [15]. Orozco-Alcala et al. suggested that inflammatory arthritis may be related to SCD [16]. Schumacher et al. published a study analyzing joint involvement in SCD. The author noted that in SCD, joint effusions were common, although transient, and did not respond to intra-articular steroids. In addition, synovial fluid was characteristically non-inflammatory and synovial biopsies lacked significant inflammatory cell infiltrates. Schumacher concluded that the etiology of effusions in SCD was likely related to bony infarcts secondary to ischemia rather than inflammatory joint disease [17].

In 1974, Espinoza et al. observed 71 patients with SCD for 18 months assessing arthropathy. They noted that the prevalence of arthritis in SCD was 46.6%, usually acute and transient, and likely related to ischemia. However, two patients in the study developed chronic synovitis with cartilage erosions. One of these patients underwent a synovial biopsy that showed mononuclear cells and plasma cell infiltration [18]. In 1984, de Ceulaer et al. followed 37 patients with sickle cell disease and non-gouty arthritis for 30 months. They also concluded that inflammatory arthritis associated with SCD is usually acute and self-limiting. They added that this transient arthritis correlates with VOC and is therefore overshadowed and under diagnosed [19].

With the wide use of auto-antibodies supporting RA diagnosis [20], and the longer life expectancy of SCD patients, more cases of SCD coexisting with seropositive RA have been reported. Three cases of SCD coexisting with RA and two cases of juvenile rheumatoid arthritis coexisting with SCD were reported [21–24]. The clinical vignettes highlight the diagnostic challenges of suspecting RA in a patient with chronic pain and inflammation, followed by the treatment dilemma.

Michel et al. performed a retrospective study analyzing connective tissue disease in patients with SCD. There were 15 patients identified with RA coexisting with SCD, with 93% having rheumatoid factor positivity, 88% having anti-citrullinated peptide antibodies and 80% had erosive joint disease [25].

A retrospective analysis conducted by our group, revealed that the prevalence of SCD coexisting with RA was 0.94%, which is similar to the prevalence of RA among the general population. Our patients, mostly from Afro-Caribbean descent, with SCD-RA were compared to age and sex matched patients with SCD and RA. The SCD-RA patients had lower hemoglobin (p<0.01) and tended to have a lower BMI, increased periarticular osteopenia, erosive arthritis, prolonged morning stiffness, increased number of hospitalizations and longer hospital stay. The SCD-RA patients were also older than the SCD-only population but younger than RA-only patient population and had been diagnosed with RA at a younger age (about 5 years earlier) than RA-only patients. SCD-RA patients had more difficulty with ADL [25]. We hypothesize that improved survival of SCD patients resulting from medical care, preventive measures such vaccinations and the introduction of hydroxyurea, have made SCD population susceptible to the development of RA that generally occurs at a more advanced age [26,27]. Furthermore, the diagnosis of RA at a younger age in SCD patients is likely the result of medical attention that these patients receive due to frequent encounters in outpatient and inpatient settings, although the role of inflammatory markers released during VOC might explain earlier RA presentation among SCD patients. However, SCD itself causes chronic musculoskeletal complaints that may delay or even obscure the diagnosis of RA. Whether SCD coexisting with RA is merely coincidental or related to shared mechanisms of inflammation will be covered in the next section.

### Pathophysiology crossroads

During VOC, damage to the RBC membrane occurs as a result of oxidation and precipitation of polymers of globin which confers the sickled RBC membrane an enhanced ability to adhere to the endothelium by exposing adhesion molecules [4,5]. Endothelial cell membrane receptors interact with a number of adhesion molecules presented by the sickled RBC. Adhesion molecules such as Vascular Cell Adhesion Molecule-1 (VCAM1), Thrombospondin, Von Willebrand factor, and Basal Cell Adhesion Molecule/Lutheran blood group (BCAM/Lu) have been studied [28]. Adhesion molecules promote leukocyte adhesion which leads to luminal narrowing and red blood cell entrapment that contribute to vaso-occlusion and inflammation. The interactions between the endothelium and adhesion molecules lead to activation of Nuclear Factor-κappa βeta (NF-κβ) which in turn drives the production of reactive oxygen species (ROS). Therefore, inflammation arises from endothelial cell activation and sickled RBC membrane damage leading to recruitment of leukocytes and platelets, which in turn release pro-inflammatory cytokines, such as Placental Growth Factor, Tumor Necrosis Factor-α, Interleukin-1 (IL-1), and Interleukin-6 (IL-6) among others. Placental Growth Factor (PlGF) has angiogenetic properties [28,29]. Furthermore, SCD patients were found to have elevated IL-1β, IL-8, IL-6, IFN-ɣ, hs-CRP, sVCAM-1 and Tissue Factor at baseline, compared to normal controls, even when not in VOC [30–32]. Ongoing episodes of VOC in SCD allows for chronic inflammation to manifest as tissue damage. Additionally, intravascular hemolysis contributes to the inflammation process as the released hemoglobin deposits on the endothelium and sub-endothelium smooth muscle layers leading to oxidative stress [31]. In addition, elevated levels of heme in bloodstream can activate innate immunity via Toll-like Receptor-4, which in turn can trigger VOC; damage-associated molecular patterns (DAMP) seem to play important role in pathogenesis of VOC in SCD as well [33]. Studies have also shown that SCD creates a pro-coagulant state, as exposed phosphatidylserine on the RBC membrane triggers thrombin generation and the extrinsic pathway of coagulation [30,34]. P-selectin [35] and E-selectin mediated neutrophil adhesion to the endothelium seems to play a pivotal role in VOC. Recruited neutrophils lead to entrapment of RBC and to lesser extent platelets also, allowing the formation of cellular aggregates that contribute to inflammation via the production of reactive oxygen species (ROS) [36–41].

When studying the pathogenesis of RA, we find that the humoral adaptive and innate immune systems are involved in the activation and perpetuation of the clinical disease in RA. The neo-epitopes presented by the inflamed synovial membrane attract plasmacytoid dendritic cells to mobilize into the synovium. Macrophages become activated via T helper 1 (Th1) cytokines like interferon (IFN-ɣ), IL-12, 15, 18, 23. Th17 cells also participate, given their production of IL-17, 21, 22 and TNF-α. IL-17 and TNF-α promote activation of fibroblasts and chondrocytes. Synovial B cells present in synovial follicles produce not only autoantibodies but also a number of chemokines such IL-6, TNF-α, and Lymphotoxin-ß that play a pathogenic role RA [9]. TNF-α activation of metalloproteinases leads to cartilage destruction while activated osteoclasts resorb bone contributing to the joint damage and erosions characteristic of RA [42]. Leukocyte adhesion plays a key role in infiltration of immune cells, playing a pivotal role in the development and perpetuation of chronic inflammation in RA [9,43,44].

Understanding the pathophysiologic overlap between SCD and RA might help explain the prevalence observed in our study, RA developing at a younger ager in SCD-RA patients and provide rationale for treatment modalities. As seen above, recurrent VOC in SCD lead to a continuous inflammatory response that shares many characteristics with RA pathophysiology. For example, as described, IL-1 and IL-6 are prominent in both SCD and RA. We postulate that recurrent elevations in TNF-ɑ, IL-1 and Il-6, as well as other pro-inflammatory cytokines in patients with SCD may increase synovitis in RA. The underlying mechanism is largely unknown at this time however, we can propose that ischemia damages SCD patient’s synovium leading to the release of synovial antigen into the circulation thus inducing autoantibody formation. This theory could explain why SCD patients were diagnosed with RA at a younger age (5 years earlier) and tended to be seropositive for RF and anti-CCP than RA only patients [25].

In addition, repeated transient ischemia and reperfusion during VOC, leads to an increase in PlGF and vascular endothelial growth factor (VEGF) which have angiogenetic properties [29,45]. Angiogenesis, an important component of inflammation in RA and pannus formation [46,48], plays a critical role in both RA and SCD which leads us to the following questions: Does increased angiogenetic cytokines in SCD predispose patients to develop RA? Will targeting angiogenesis or endothelial adhesion molecule expression in RA have an impact on the management of SCD in this patient population? Mechanistic questions remain to be answered in future research.

**Figure 1:**
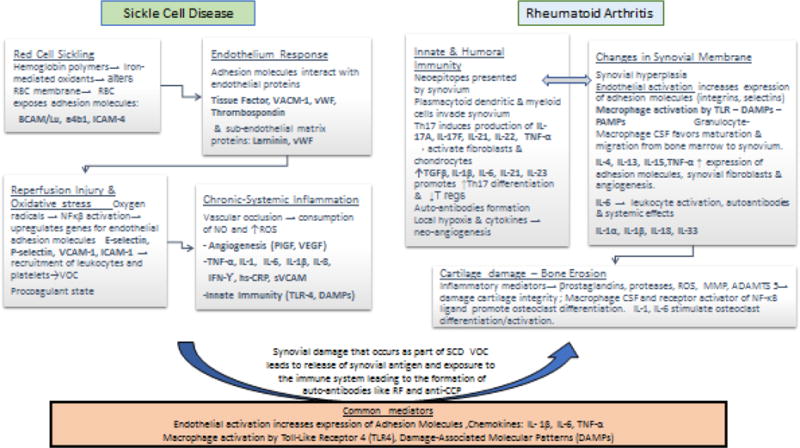
Pathogenesis of Sickle Cell Disease and Rheumatoid Arthritis: Postulated Mechanism and Common Mediators Involved [3,9].

Nitric oxide (NO), a free radical produced by the vascular endothelium nitric oxide synthase from arginine, acts as a potent vasodilator, inhibits platelet aggregation and inhibits adhesion of platelets to the vascular endothelium. In patients with SCD, hemolysis leads to the release of free hemoglobin that not only binds to NO but also generates free superoxide radicals and disrupts endothelial NO synthase functioning [49,50]. This leads to decreased bioavailability of NO and decreased vasodilation producing a paradoxical compensatory increase of NO expression in tissues despite endothelial dysfunction [51]. ROS which originate as a result of mitochondrial oxidative phosphorylation in response to foreign chemicals, bacteria and cytokines, are also implicated in the pathogenesis of RA by leading to increased inflammation and synovitis. RA serum and synovium have been shown to have higher activity of oxidative enzymes and lower levels of anti-oxidants. Oxidative damage of cartilage, extracellular collagen and DNA have been described and NO has been found to be upregulated in RA synovial tissue [52]. We proposed that ROS generated during VOC can contribute to the inflammatory process of SCD-RA, leading to a more severe disease course (Figure 1).

In summary, both processes involve endothelial activation which augments the expression of adhesion molecules that play a role in the initial steps of VOC in SCD and leukocyte trafficking in RA; elevation in serum levels of chemokines such as IL-1β, IL-6, TNF activate white blood cells, endothelial cells, and synovial fibroblasts, induce enzymatic matrix damage and activate osteoclasts; production of ROS; macrophage activation by Toll-Like Receptor 4 (TLR4) and Damage-Associated Molecular Patterns (DAMPs) have been also described in the pathogenesis of both comorbidities [3,9]. We postulate that ischemic changes resulting from SCD VOC damages the synovium with subsequent release of synovial antigen into the circulation. The humoral and innate immune systems will be activated leading to autoantibody formation such as Rheumatoid factor and anti-citrullinated protein antibody.

ADAMTS5 a disintegrin and metalloproteinase with thrombospondin motifs 5, a4b integrin on platelet surface that mediates adhesion to collagen, BCAM/Lu basal cell adhesion molecule Lutheran blood group, CSF colony stimulating factor, DAMPs Damage-associated molecular patterns, hs-CRP high sensitivity C reactive protein, ICAM-1 Intercellular adhesion molecule 1, IL interleukin, INF interferon, NF-κβ, Nuclear Factor-Кappa Beta, PlGF platelet growth factor RBC red cell membrane, RF Rheumatoid Factor, ROS reactive oxygen species, sVCAM serum vascular adhesion molecule, TLR-4 Toll Like Receptor 4, TGF-β Tissue growth factor β, TNF-α tumor necrosis factor α, T regs Regulatory T cells, MMP matrix metalloproteinases, VACM-1 vascular cell adhesion molecule, VEGF vascular endothelium growth factor, VOC vaso-occlusive crises, vWF Von Willebrand Factor.

### Treatment

The treatment of rheumatoid arthritis in patients with coexisting sickle cell disease presents a unique challenge, as the use of immunosuppressive agents is contingent upon first making the diagnosis of rheumatoid arthritis [20,53]. The main diagnostic difficulty is distinguishing musculoskeletal complaints related to SCD, such as pain from VOC, from chronic inflammatory arthritis. Other manifestations of SCD are transient arthritis associated with effusions, avascular necrosis, and asplenia, making SCD patients vulnerable to infections [54,55]. These patients therefore require a thorough history, physical exam, laboratory tests, and appropriate imaging prior to treatment with immunosuppressive agents.

Once other causes of arthritis have been ruled out and the patient meets criteria for RA [20], the choice of medications becomes controversial. Corticosteroids are universally accepted as fast-acting immunosuppressive agents that plays a major role in inflammatory disorders; however, there is anecdotal evidence that it may trigger sickle cell crisis. Gladman et al. reported 2 cases of patients developing acute sickle cell crises after receiving intra-articular injection of methylprednisolone [56]. There are several other case reports demonstrating severe sickle cell crises following corticosteroid use [57–60]. In contrast to the anecdotal evidence, Griffin et al showed that a short course of high-dose corticosteroids decreased duration of severe pain episodes and was not associated with adverse reactions [61]. A small retrospective study showed that long term steroid use in patients with SCD was associated with increased sickling crises [61,62].

Another consideration is the interaction that the anti-rheumatic drugs will have on SCD patients managed with hydroxyurea. Hydroxyurea became standard of care for SCD when it was demonstrated hydroxyurea increases fetal hemoglobin (HbF) and decreases the frequency of VOC. Hydroxyurea also lowers white cell count, decreases reticulocyte count and LDH levels, all of which impacts the development of VOC complications, organ damage and early death [63,64].. Hydroxyurea also decreases total count of CD4+ T cells, although it remains within normal limits [65], and there are isolated case reports of opportunistic infections developing in patients with SCD on hydroxyurea [66]. It is not known if patients with SCD-RA on hydroxyurea should have limitations with respect to using additional immunosuppressive agents [67], or if they have less disease activity of RA as autoreactive CD4+ T cells are involved in the immunopathogenesis of RA [68]. Based on our review of the literature, steroids should only be used sparingly in RA patients with coexisting SCD. When steroids are used it should be with caution and in short courses only.

Early initiation of disease modifying antirheumatic drugs (DMARDS) are standard of care for patients with RA [53]. The concomitant use of hydroxychloroquine along with hydroxyurea and didanosine was studied by Paton and Aboulhab in 1995 in a cohort of HIV patients who received the drug combination for 144 weeks [69]. The study was designed to test the antiviral efficacy and safety of the triple therapy. Hydroxychloroquine and hydroxyurea were tolerated well and provided long term control in viral replication. No ocular adverse events were reported. The FDA has received a small number of reports of concomitant anti-malarial and hydroxyurea usage; adverse effects have been related to liver function tests and cell blood counts abnormalities [70].

The use of sulfasalazine in the treatment of patients with SCD and RA has not been reported; however, Solovey et al. discussed the use of sulfasalazine in patients with SCD on folic acid. The use of sulfasalazine significantly reduced the expression of VCAM, intercellular adhesion molecule 1 (ICAM-1), and E-selectin in circulating endothelial cells and caused downregulation of NF-κβ, a transcription factor that promotes gene expression of pro-adhesive molecules on endothelium [71]. It is unknown, however, whether sulfasalazine would have any interaction with hydroxyurea.

In regards to the safety of Methotrexate (MTX) usage in SCD patients, Brandalise S, et al. conducted a pilot study in SCD patients on hydroxyurea, to examine the impact of MTX on VOC. The authors observed that the frequency of VOC did not decrease; however, avascular necrosis pain diminished, bringing gain in physical functioning, which suggested some benefit with the combination of MTX and hydroxyurea. Low platelet counts were observed as adverse effects with the double therapy [72]. Furthermore, patients with SCD will have compensatory reticulocytosis which can be impaired with MTX. Nevertheless, when indicated, MTX should be used with close monitoring of cell counts including reticulocytes and liver function tests. Leflunomide administration would be often limited by the abnormal liver tests associated with SCD and azathioprine by the increased risk of myelosuppression (Table 1).

There is almost no literature regarding the use of biologics in the management of RA in SCD. Cordner et al. suggests that TNF-α blockers appear to be a logical treatment choice given their efficacy in RA and TNF-α’s role in expression of adhesion molecules in the vascular endothelium [73]. Michel et al. had a cohort of 4 RA-SCD patients successfully treated with TNFα inhibitors [74]. Three patients responded within 8 weeks of anti-TNF initiation but one patient failed to respond and was switched to rituximab. At 3 months following rituximab therapy, the patient had an ACR20 response and neither VOC nor infections were reported [74].

Using biologics to downregulate the vaso-occlusive pathway of SCD is a possible approach given the common mediators that are implicated in the pathogenesis of SCD and RA as previously described. Ataga et al. published earlier this year the results of the SUSTAIN trial, using crizanlizumab, an antibody against the endothelial and platelets adhesion molecule P-selectin for treatment of SCD [35]. The P-selectin inhibitor was studied in a double blind, randomized placebo-controlled fashion in 198 SCD patients. Low dose and high dose crizanlizumab groups were observed for 52 weeks and those patients already on hydroxyurea were continued on the drug. The annual rate of hospitalization, number of VOC, complicated and uncomplicated events were recorded. The group of SCD patients receiving the higher dose of crizanlizumab had a 62.9% lower rate of uncomplicated crises. The incidence of adverse events and serious adverse events were similar in the crizanlizumab and placebo groups. Antibodies against crizanlizumab did not develop in any of the patients [35]. The SUSTAIN study results open the possibility of a novel approach for the management of SCD-RA patients.

Statins, recognized to improve vascular function by decreasing inflammation and endothelial activation, have also been explored in animal models with SCD and pulmonary hypertension [32]. Hoppe in 2011 treated 26 children with SCD with 20 mg and 40mg of simvastatin. Plasma nitric oxide metabolites (NOx), C-reactive protein (CRP), IL-6, VCAM-1, VEGF and tissue factor were measured before and after the 21-day trial. Hydroxyurea was continued throughout. Simvastatin was well tolerated and safe, levels of NOx increased while CRP and IL-6 levels dropped in a dose related fashion, which suggested a potential therapeutic role for statins in disease entities where endothelial activation plays a pivotal role.

Finally, we need to stress the importance of vaccination against encapsulated bacteria in this patient population. In addition, patients should undergo appropriate tuberculosis screening before anti-TNF, and glucose-6-phosphate dehydrogenase testing when considering a trial of hydroxychloroquine. As recommended by American College of Rheumatology, patients started on DMARDs should have blood cell counts and chemistries including liver function as per recommended intervals [53].

## Conclusion

There is a scarcity of literature regarding the coexistence of rheumatoid arthritis and sickle cell disease. Furthermore, the severity of the musculoskeletal symptoms in SCD can delay the diagnosis of RA. It is possible that the underlying inflammatory mechanism of SCD and RA may worsen the clinical manifestations of each disease affecting the prognosis.

Our data suggest the SCD-RA patients develop more erosions and periarticular osteopenia, are younger at the age of presentation and tended to be more seropositive for autoantibodies compared to RA only population. This raises the possibility that ischemia from SCD might lead to synovial damage and induction of autoimmunity earlier compared to patients without SCD (RA only group).

Additionally, treatment decisions are complicated by occurrence of VOC, hemolysis, asplenia and adverse reactions associated with currently available therapies. Further research is needed to establish the characteristics and the clinical course of RA among SCD patients, disease activity, radiological findings, impact on the quality of life and prognosis, as well as treatment options for these patients that could target early steps in the pathogenetic cascade of chronic inflammation of these two debilitating diseases.

## Figures and Tables

**Table 1:** Available Therapies for Rheumatoid Arthritis and Potential Effects in SCD Patients.

Drug Class/Name	Mechanism of action	Reported uses in SCD patients	Reported possible interaction with hydroxyurea
Glucocorticoids	Suppress transcription factors: AP-1 and NF-κβ. Suppress expression of inflammatory genes encoding T cell growth factors (IL-2, IL-4, IL-15, IL-17, and IFN-γ). Reduce expression of genes encoding COX-2, inducible nitric oxide synthase, and ICAM-1 Increase expression of genes encoding anti-inflammatory molecules (IL-10 and IL-1 type 2 decoy receptor).	For hyper-hemolytic crisis and acute chest syndrome. Adverse events: Rebound pain attacks, Severe pain episodes and stroke after administration of steroids for rheumatoid arthritis due to increased leukocyte counts induced by steroids [56–62].	No data reported
Methotrexate	Blocks DNA synthesis and hence cellular proliferation but also induces release of adenosine. This inhibits chemotaxis of polymorph neutrophils and release of critical cytokines as TNF-alpha and Interleukins 6 and 8. such	Vaso-occlusive crisis MTX did not reduce acute VOC frequency/intensity; decreased and it chronic pain led to improvement in quality of life.	Three patients with previous history of hydroxyurea-induced hematological toxicity developed blood low platelet counts while receiving simultaneously MTX and hydroxyurea [72].
Sulfasalazine	Reduces endothelial activation by inhibiting NF-κβ, the transcription factor that promotes expression of genes for a number of pro-adhesive and pro-coagulant molecules on endothelium.	Pilot study in three SCD only patients. Sulfasalazine reduced circulating endothelial cells expression of VCAM, ICAM, and E-selectin, but it did not reduce expression of Tissue Factor [71]	No data reported
Anti-TNF	TNF receptor blockade. Trans-membrane TNF blockade that interferes with apoptosis, cytotoxicity, cell migration and release of cytokines.	Anecdotal reports on the use of anti-TNFα on SCD-RA [73,74].	No data reported

APS-1: Activation Protein 1; COX-2: Cyclo-Oxygenase-2; DNA: Deoxyribonucleic Acid; ICAM-1: Intercellular Adhesion Molecule 1; IL: Interleukin; INF: Interferon; MTX: Methotrexate; NF-κβ: Nuclear Factor-Кappa Beta; PlGF: Platelet Growth Factor: RBC: Red Cell Membrane; ROS reactive oxygen species, sVCAM serum vascular adhesion molecule, TL-4 toll like receptor 4, TGF-β: Tissue Growth Factor β; TNF: Tumor Necrosis Factor; VACM-1: Vascular Cell Adhesion Molecule; VEGF: Vascular Endothelium Growth Factor; VOC: Vaso-Occlusive Crises.

## Abbreviations

SCD: Sickle Cell Disease
RA: Rheumatoid Arthritis
RBC: Red Blood Cells
HbS: Hemoglobin S
VOC: Vaso-Occlusive Crises
HLA-DRB1: Human Leukocyte Antigen-DRB1
TNFR: Tumor Necrosis Factor Receptor
